# Lineage-associated Human Divergently-paired Genes Exhibit Structural and Regulatory Characteristics

**DOI:** 10.1093/gpbjnl/qzaf058

**Published:** 2025-06-26

**Authors:** Guangya Duan, Sisi Zhang, Bixia Tang, Jingfa Xiao, Zhang Zhang, Peng Cui, Jun Yu, Wenming Zhao

**Affiliations:** National Genomics Data Center, China National Center for Bioinformation, Beijing 100101, China; Beijing Institute of Genomics, Chinese Academy of Sciences, Beijing 100101, China; University of Chinese Academy of Sciences, Beijing 100049, China; National Genomics Data Center, China National Center for Bioinformation, Beijing 100101, China; Beijing Institute of Genomics, Chinese Academy of Sciences, Beijing 100101, China; National Genomics Data Center, China National Center for Bioinformation, Beijing 100101, China; Beijing Institute of Genomics, Chinese Academy of Sciences, Beijing 100101, China; National Genomics Data Center, China National Center for Bioinformation, Beijing 100101, China; Beijing Institute of Genomics, Chinese Academy of Sciences, Beijing 100101, China; University of Chinese Academy of Sciences, Beijing 100049, China; National Genomics Data Center, China National Center for Bioinformation, Beijing 100101, China; Beijing Institute of Genomics, Chinese Academy of Sciences, Beijing 100101, China; University of Chinese Academy of Sciences, Beijing 100049, China; Shenzhen Branch, Guangdong Laboratory for Lingnan Modern Agriculture, Genome Analysis Laboratory of the Ministry of Agriculture and Rural Affairs, Agricultural Genomics Institute at Shenzhen, Chinese Academy of Agricultural Sciences, Shenzhen 518120, China; National Genomics Data Center, China National Center for Bioinformation, Beijing 100101, China; Beijing Institute of Genomics, Chinese Academy of Sciences, Beijing 100101, China; University of Chinese Academy of Sciences, Beijing 100049, China; National Genomics Data Center, China National Center for Bioinformation, Beijing 100101, China; Beijing Institute of Genomics, Chinese Academy of Sciences, Beijing 100101, China; University of Chinese Academy of Sciences, Beijing 100049, China

**Keywords:** Divergently-paired gene, Evolutionary conservation, Divergent promoter, Co-expression regulation, Gene cluster

## Abstract

Divergently-paired genes (DPGs) are minimal co-transcriptional units of clustered genes, representing over 10% of human genes. Our previous studies have shown that vertebrate DPGs are highly conserved compared to those from invertebrates. Three critical questions remain: (1) which DPGs are conserved across vertebrates, especially among mammals and primates? (2) to what extent and precision do these paired promoters share their sequences mechanistically and stringently? and (3) how are human DPGs distributed over selected primate lineages, and what are their possible biological functional consequences? There are 1399 human DPGs (approximately 12% of all human protein-coding genes), of which 1136, 1118, 925, and 830 human DPGs show conservation when compared to selected primates, mammals, avians, and fish, respectively. DPGs are not only functionally enriched toward direct protein–DNA interactions and cell cycle synchronization, but also exhibit lineage association, narrow in principle toward synchronization of certain core molecular mechanisms and cellular processes. Second, the inter-transcription start site (inter-TSS) distances affect both co-expression strength and disparity between the two genes of a DPG. Finally, among primates, human-associated DPGs exhibit diversification in their co-expression patterns and gene duplication events, and are obviously involved in neural development. Comparing high-quality human reference genomes from European (T2T-CHM13) and Chinese (T2T-YAO) populations, we identified 55 and 357 DPGs unique to the former and the latter, respectively. Our findings offer novel insights into the regulatory characteristics between neighboring genes and their structure–function selection among functionally conserved gene clusters.

## Graphical abstract

**Figure:**
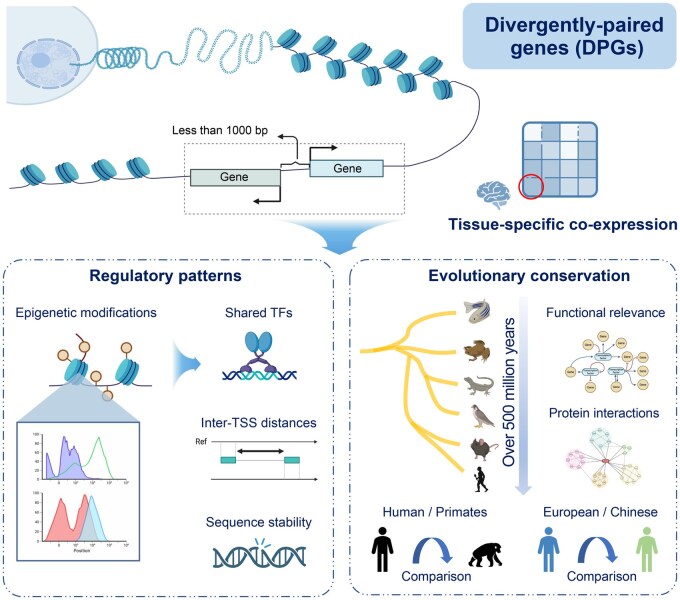

## Introduction

In prokaryotes, gene clusters, commonly referred to as operons, represent a prevalent organizational formality where numerous non-randomly aggregated genes contribute to a singular function or phenotype [1,2]. Eukaryotes also exhibit a non-random distribution of certain gene families or classes, forming distinct structural configurations from primary sequences to three-dimensional topological structures of chromatin. Paired genes, as a form of minimal gene clusters, manifest in three configurations: divergently-paired, tandemly-paired, and convergently-paired [3,4]. Such genomic architectures are prevalent in diverse animal and plant lineages, underscoring their common manifestation [5–9]. It has been observed that over 10% of the human genes, *i.e.*, divergently-paired genes (DPGs), have a bidirectional orientation separated by less than 1000 bp [4,10]. Such a spacing constraint suggests advantages in sharing promoter elements and synchronized gene expression or co-expression, which may be functionally selected over an evolutionary time scale [6,11].

Comparative genomic analyses have indicated a high degree of conservation and stability of this gene-pairing architecture among vertebrate genomes [5,12,13]. For instance, there are more fully-conserved DPGs among species of vertebrate lineages, when compared with those of the invertebrates as represented by insects [3]. Unlike the unidirectional model, DPGs are not formed from random chromosomal rearrangement events but show functional relevance across eukaryote genomes [14–16]. The formation of DPGs, by reorganizing existing genes, often leads to novel co-regulated transcription, representing a mechanism to acquire new components beneficial to species fitness [6,17,18]. Hence, the identification and study of lineage-associated (for association within or among lineages) and species-specific (for uniqueness in species) DPGs are essential in understanding diverse gene and genome evolution processes.

The detailed regulatory mechanisms of DPGs have recently been readdressed. The sequence-space-limited organization of DPG promoters places the two neighboring genes under pressure to share their regulatory elements at least in part, offering an opportunity to scrutinize how *cis-*elements and *trans*-elements interact in molecular detail at the transcriptional level [19]. Several regulatory mechanisms, including epigenetic signals, have been identified, such as RNA polymerase II (RNAPII) occupancy and histone modifications of H3K4me2, H3K4me3, and H3ac, which are overrepresented at bidirectional promoters [20–22]. Additionally, the nucleosome-depleted regions (NDRs) between paired genes bring an optimal condition for co-regulation [11,23]. Furthermore, bidirectional promoters, commonly found among DPGs, often lack typical TATA boxes but possess high GC content and enrichment of CpG islands [4,24]. Moreover, they display distinctive sequence signatures characterized by a mirror composition, where Gs and Ts prevail on one side, and Cs and As dominate the opposing sides [25]. The DPG-associated promoter sequences also influence transcription factor (TF) binding, and a small set of TF motifs appear to be overrepresented within the shared space [20].

As sequencing technology has advanced greatly in recent years, the quantity and quality of genomic data in various forms and from diverse sources have been increasing, allowing for in-depth studies, including orthologous relationships between genes, functional annotations, epigenetic modifications, RNA sequencing (RNA-seq) data, and TF motifs. Leveraging multiple-level high-quality datasets from diverse sources, we explored the evolutionary history and trajectory of DPGs across vertebrate lineages in a human-centric way and conducted a comprehensive comparative analysis based on primate data collections. In addition, taking advantage of recently-released human reference genomes, two of which are attributed to telomere-to-telomere (T2T) quality, *i.e.*, T2T-CHM13 of European origin [26] and T2T-YAO [27] of the first representative tailored to Chinese populations, we examined not only genomic disparities of DPGs across vertebrate lineages but also those between the human populations. Furthermore, we analyzed the regulatory mechanisms underlying co-expressed DPGs, including genetic and epigenetic mechanisms, regulatory patterns of co-expression, shared TFs within inter-transcription start site (inter-TSS) sequences, and other structural and functional features, thereby enhancing our understanding of this important structural arrangement.

## Results

### Human DPGs exhibit complex lineage associations

**Figure 1:**
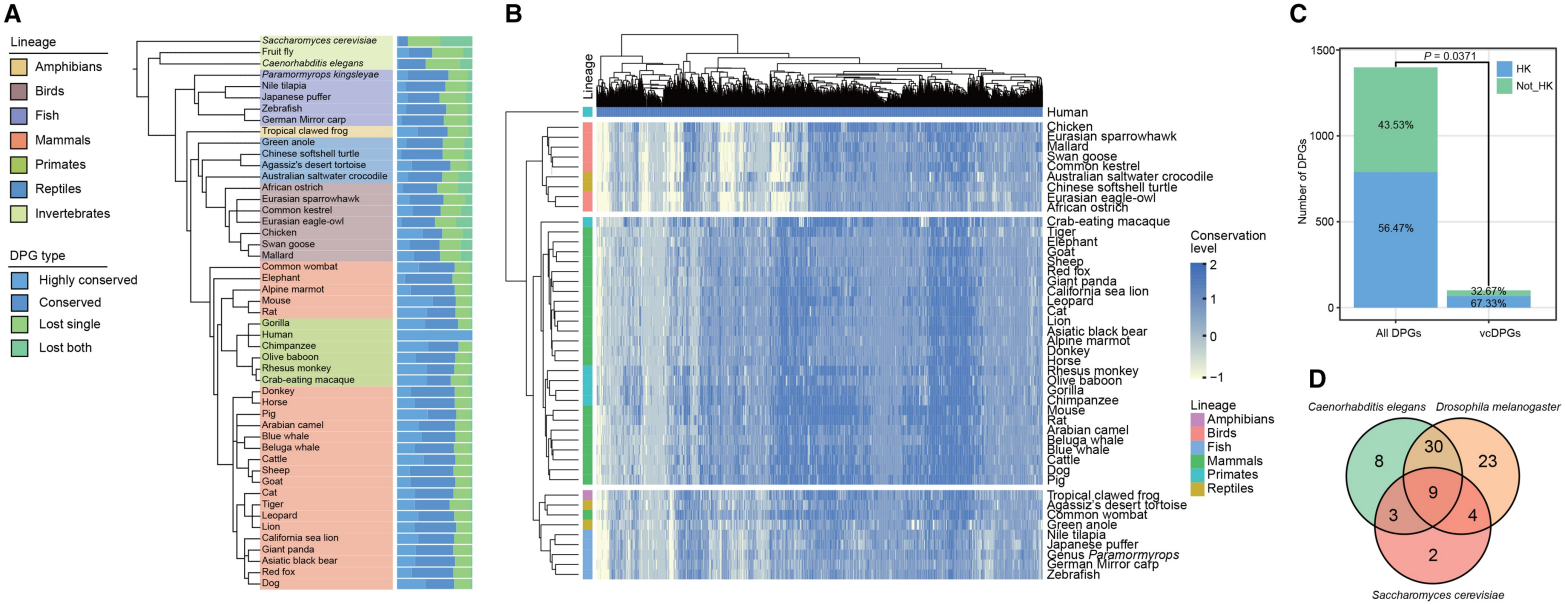
Four conservation forms of human DPGs across diverse vertebrate lineages **A**. Fractions of the four gene-defined and orientation-defined forms of conserved DPGs from different species. The organization is based on phylogenetic tree originating from NCBI taxonomy. The detailed classification method is shown in Materials and methods. **B**. Heatmap showing the conservation of human DPGs in 45 other vertebrates, where darker colors indicate higher conservation. The values of 2, 1, 0, and −1 correspond to the four conservation types, representing a conservation scale from high to low. The columns and rows are grouped by two-way hierarchical clustering. **C**. Proportion of DPGs containing HK genes in all human protein-coding DPGs and in the 101 vcDPGs. **D**. A diagram illustrating the conservation of vcDPGs in three selected eukaryotic multi-cellular and unicellular species: *Drosophila melanogaster*,* Caenorhabditis elegans*, and *Saccharomyces cerevisiae*. DPG, divergently-paired gene; HK, housekeeping; vcDPG, vertebrate-conserved DPG; NCBI, National Center for Biotechnology Information.

Utilizing spacing-based criteria (inter-TSS distances < 1000 bp), we started our analysis with a set of 1399 human protein-coding DPGs (Table S1). To investigate their evolutionary conservation patterns across several vertebrate lineages, we selected, aside from human, 45 species from 6 distinct lineages for further comparative analysis (**Figure 1**; Table S2). Four conservation categories were defined to quantify the DPG conservation levels (see Materials and methods for details). Among the lineages and species, the four conservation categories are in similar proportions, varying based on sequence identities relative to the human genes, but their precise numbers may be difficult to define due to the quality of their genome sequences. For instance, gorillas and chimpanzees show the highest numbers of highly conserved human DPGs, which is to be expected, but appear to show fewer conserved DPGs when compared to those of the mouse due to the better murine genome assembly. Three invertebrate species, *Drosophila melanogaster*,* Caenorhabditis elegans*, and *Saccharomyces cerevisiae* were also included in our analysis as representative outgroups (Figure 1A). A hierarchical clustering mapped the species based on their sequence-based similarity distance in each row and displayed them as three major groups: the mammals are stand-alone, fishes and amphibians are grouped together, and avians form the third group. The reptiles split into either avians or the lower vertebrate groups, and such a deviation from expectation is attributable to two reasons at least: one, the small number of reptilian species used for the analysis, and the other, the fact that many DPGs are species-associated and lineage-associated, which are partly convergently evolved and selected for their functional significance (Figure 1B). Conversely, most lineage-associated DPGs appear to follow a divergent trend, where the 1399 human protein-coding DPGs share 1136, 1118, 830, and 925 with other primates, mammals, birds, and fish, respectively. We even have 101 vertebrate-conserved DPGs (vcDPGs) over all 46 selected species for this study.

We also looked into two other interesting aspects, partitioning between housekeeping (HK) and tissue-specific (TS) functions and conservation across distinct lineages. Using a list of HK genes as benchmarks from a previous study [28], we found that DPGs are slightly HK-gene enriched, harboring 56.47% of all and 67.33% of vcDPGs (Figure 1C), whereas the general partition of HK and TS genes is half-and-half [29]. There are 66, 50, 18, and 9 DPGs conserved in *Drosophila melanogaster*, *Caenorhabditis elegans*, and *Saccharomyces cerevisiae*, and all three species, respectively, showing conservation of these gene pairs even among invertebrates (Figure 1D; Table S3).

### Functional relevance of vcDPGs

**Figure 2:**
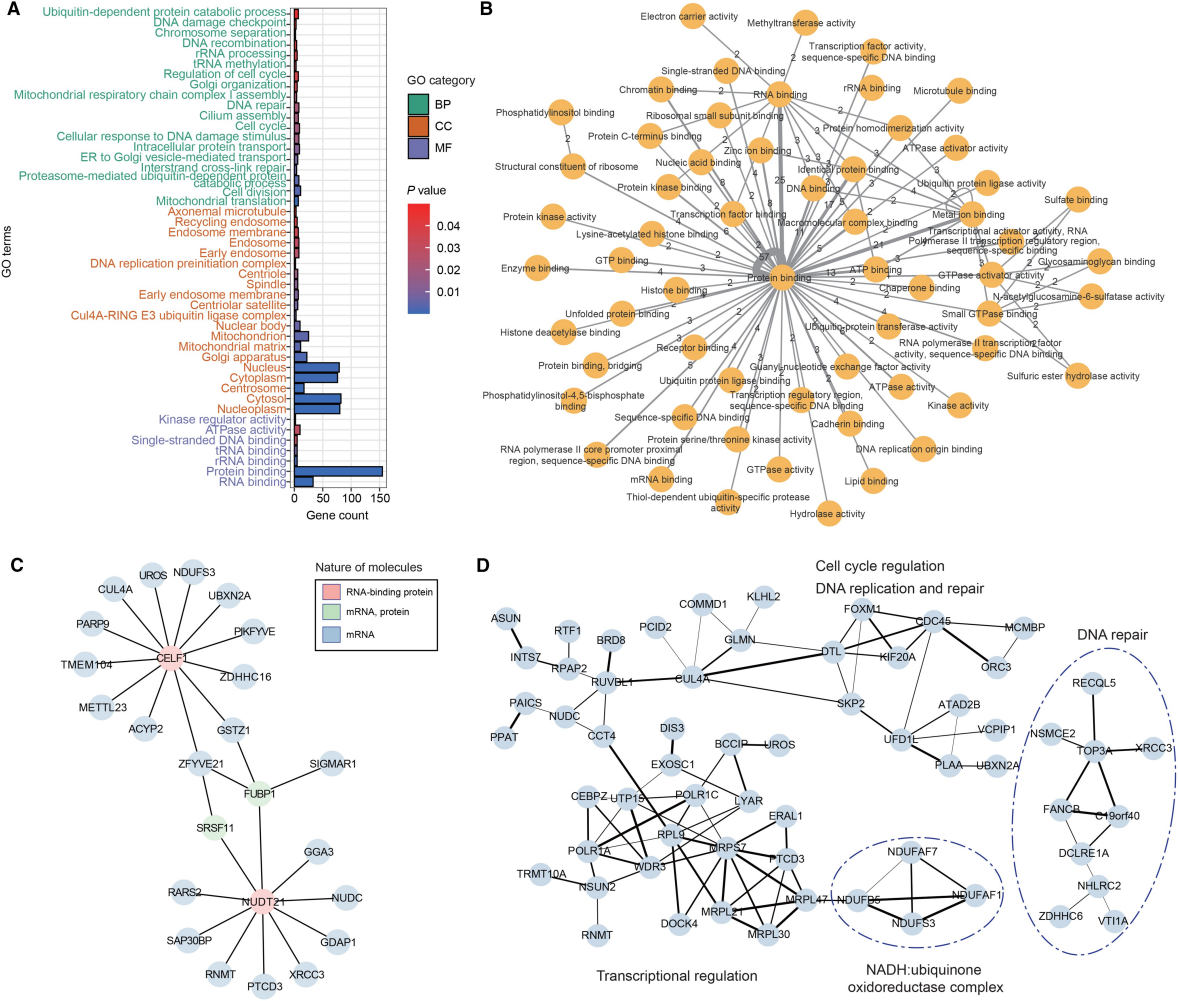
Functional interactions of vcDPGs **A**. Functional enrichment of GO annotations of the 101 vcDPGs obtained from DAVID. Bars are color-coded based on *P* values, and GO terms are color-coded based on their categories. **B**. Functional correlation network of vcDPGs, filtered with a minimum correlation count greater than 1. Nodes represent GO terms in the MF category, and edges denote functional correlations between vcDPGs. Circles indicate vcDPGs with both genes sharing the same GO term. The numbers on edges indicate DPG count associated with that correlation. Most vcDPGs have functional correlations with the GO term “protein binding”, as indicated by the thick circle. **C**. Protein–RNA interaction network of vcDPGs, filtered with a high-confidence threshold using data obtained from the RNAInter database. The nature of the molecules is color-coded. **D**. A PPI network of vcDPGs. The data are filtered based on a medium-confidence threshold, preserving the primary clusters. All interactions are listed in Table S4. The clusters are composed of two major groups, one on the left and a separate group on the right. GO, Gene Ontology; BP, biological process; MF, molecular function; CC, cellular component; PPI, protein–protein interaction; rRNA, ribosomal RNA; tRNA, transfer RNA; ER, endoplasmic reticulum; mRNA, messenger RNA; DAVID, Database for Annotation, Visualization and Integrated Discovery; RNAInter, RNA interactome.

The functional relevance of DPGs has long been known as they must follow lineage-associated characteristics defined based on evolutionary principles and criteria [30,31]. Since DPGs stand out as a unique class of co-expressed genes for genome-wide functional and mechanistic analyses, we decided to take a subgroup of them as an example for in-depth exploitation, and thus the choice of vcDPGs. Gene Ontology (GO) enrichment analysis suggests “binding” functions in the molecular function (MF) category, such as protein–RNA binding. In the category of biological process (BP), the enrichment appears to relate to DNA repair, cell cycle, and intracellular protein transport. As to the cellular component (CC) category, what is enriched includes the nucleus, cytoplasm, cytosol, and nucleoplasm (**Figure 2**A). These results suggest that vcDPGs, belonging to the most conserved group of genes over 500 million years of vertebrate evolution [32,33], are most likely to be associated with certain fundamental binding functions that coordinate or synchronize the timing of cell-cycle-related processes through direct interactions among proteins, RNAs, and DNA. In other words, these proteins tend to play regulatory roles instead of evolving into functional proteins enriched in particular pathways.

To provide further insight, we investigated the MF category based on functional network analysis, which highlights 57, 21, 21, and 11 binding details as protein binding, protein–RNA binding, protein–metal ion binding, and protein–DNA binding, respectively (Figure 2B). From this list of genes, we learned that these bindings are not only physical interactions between or among certain proteins but are mostly involved in catalytic activities, such as phosphorylation and other post-translational modifications (PTMs). We constructed both RNA–protein interaction and protein–protein interaction (PPI) networks for further illustration. For instance, our RNA–protein interaction network analysis reveals that CUGBP Elav-like family member 1 (CELF1) and Nudix hydrolase 21 (NUDT21), both functioning as RNA-binding proteins, are involved in non-coding RNA (ncRNA) and messenger RNA (mRNA) processing, respectively, and are partitioned into two distinct yet related network clusters (Figure 2C). Surprisingly, their DPG counterparts encode protein tyrosine phosphatase, mitochondrial 1 (PTPMT1) and 2-oxoglutarate and iron-dependent oxygenase domain containing 1 (OGFOD1), respectively, which are enzymes of different cellular functions. Similarly, most DPGs involved in protein–ion interactions are predominantly related to zinc binding, with a few exceptions mostly involved in interactions with bivalent ions, such as calcium, copper, and magnesium ions (Table S4). These highly conserved vcDPGs appear diverse in functions but may orchestrate the formation of protein-prosthetic complexes that are essential or cellular housekeeping processes.

The final examples are composed of two PPI clusters; both are related to DNA repair and also linked with RNAPII, mitochondrial ribosomal proteins, and subunits of ubiquinone oxidoreductase (Figure 2D; Table S4). To investigate direct interactions between the gene pairs among these DPGs, we compared the collection to 101 randomly selected gene pairs. In such a sampling test, only 1 out of the 101 randomly selected pairs exhibited direct protein interactions, whereas 41 out of the vcDPG pairs did so. This result suggests that both the spatial proximity of DPGs and their coordinated expression regulation contribute to their functions, mostly selected for direct molecular interactions (*P* = 1.347E−13, Chi-squared test).

### Human DPGs show distinct tissue specificity and co-expression patterns

**Figure 3:**
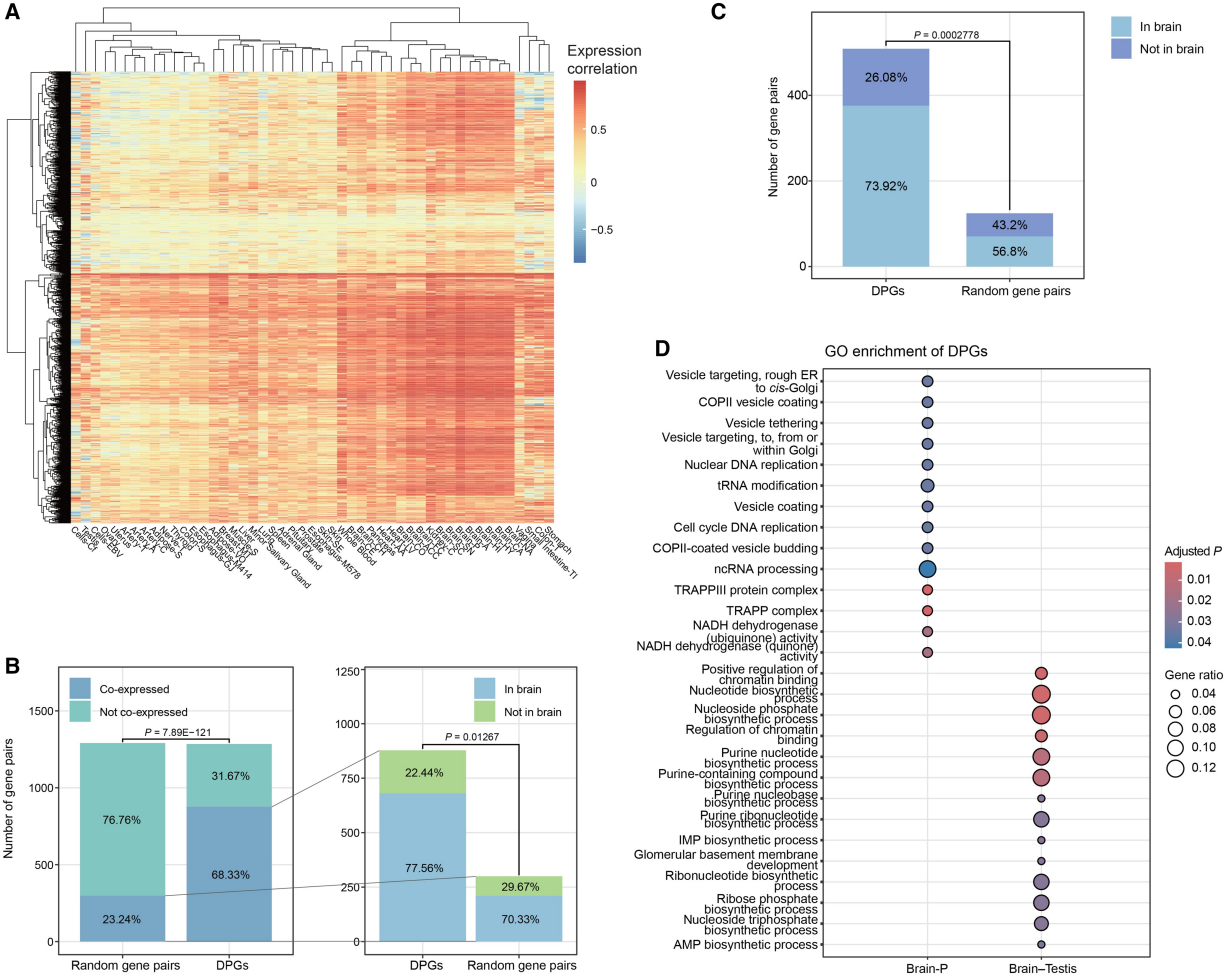
Tissue specificity and co-expression of DPGs **A**. Correlation coefficients between expression levels of DPGs across all tissues. The rows of DPGs and columns of tissues are grouped by two-way hierarchical clustering. Tissues are labeled with simplified names, and the full names are detailed in Table S5. **B**. Number and proportion of co-expressed DPGs compared to random gene pairs. A zoomed-in view of the co-expressed proportions shows the number and proportion of DPGs co-expressed in brain-related tissues compared to random gene pairs. **C**. Comparison after removing HK genes from both the DPGs and random gene pairs. Chi-squared test was used to compute the difference in (B and C). **D**. Functional enrichment of DPGs co-expressed in the Brain-P tissue and DPGs co-expressed in both brain and testis tissues. ncRNA, non-coding RNA; AMP, adenosine monophosphate; IMP, inosine monophosphate; Brain-P, brain-putamen (basal ganglia); NADH, nicotinamide adenine dinucleotide.

Although co-expression of DPGs has been extensively observed [31,34], their tissue specificity and the degree of such a coordinated cellular activity need further examination at a single DPG level. We conducted co-expression analysis for all 1399 DPGs across 51 tissues (**Figure 3**A). We observed that DPG expression in brain-related tissues manifests higher co-expression coefficients as expected for TS genes, and some DPGs show opposing expression trends in different tissues. For example, the expression of the *GSKIP–ATG2B* pair shows a positive correlation of 0.87 in brain putamen (basal ganglia) and heart-left ventricle tissues, but a evident negative correlation of −0.6 in colon-transverse and vagina tissues (Figure S1A). Conversely, the colon has the fewest co-expressed DPGs, totaling only 6. Compared with 1399 randomly selected gene pairs from the human genome, DPGs exhibit a significant co-expression effect (*P* = 7.89E−121, Chi-squared test; Figure 3B), particularly in brain-related tissues (*P* = 1.267E−2, Chi-squared test; Figure 3B). After removing HK genes from the DPG set, the result becomes more pronounced (*P* = 2.778E−4, Chi-squared test; Figure 3C), highlighting that both tissue specificity and co-expression are essential characteristics of DPGs.

A total of 14 co-expressed DPGs are ubiquitously found in all 51 tissues, such as the *DTX3L–PARP9* and *TMEM176A–TMEM176B* pairs. These gene pairs are functionally enriched in essential biological processes, including structural integrity (*e.g.*, basement membranes and collagen), protein homeostasis (*e.g.*, proteasome function), and immune response regulation (*e.g.*, dendritic cells and virus defense) [Benjamini*–*Hochberg (BH) adjusted *P* < 0.05; Figure S1B; Table S6]. Others are TS, co-expressed in brain putamen (basal ganglia) tissues and enriched in ncRNA processing, mitochondrial functions, and coat protein II (COPII) vesicle coating. Interestingly, a subset of DPGs is co-expressed in both testis and brain tissues, enriched in nucleotide biosynthetic processes (BH adjusted *P* < 0.05; Figure 3D). The PPI networks for these brain–testis co-expressed DPGs (Figure S1C) reveal several functional regulatory clusters: mitochondrial function and energy production, where proteins such as ATP5F1C, NDUFA1, and NDUFB11 are essential for cellular energy metabolism; proteasomal adaptations, where interactions among PSMB9, PSMB8, and PARP9 suggest adaptive mechanisms for protein turnover and immune response regulation, with PSMB8 and PSMB9 involved in antigen processing; and immune system and membrane transport, where interactions within the MS4A family (*e.g.*, MS4A4A and MS4A6A) imply evolutionary adaptations in immune cell signaling and pathogen recognition. These results suggest that tissue specificity and co-expression of lineage-associated DPGs should be considered separately with special attention.

### Highly co-expressed DPGs entertain inclusive regulatory mechanisms

**Figure 4:**
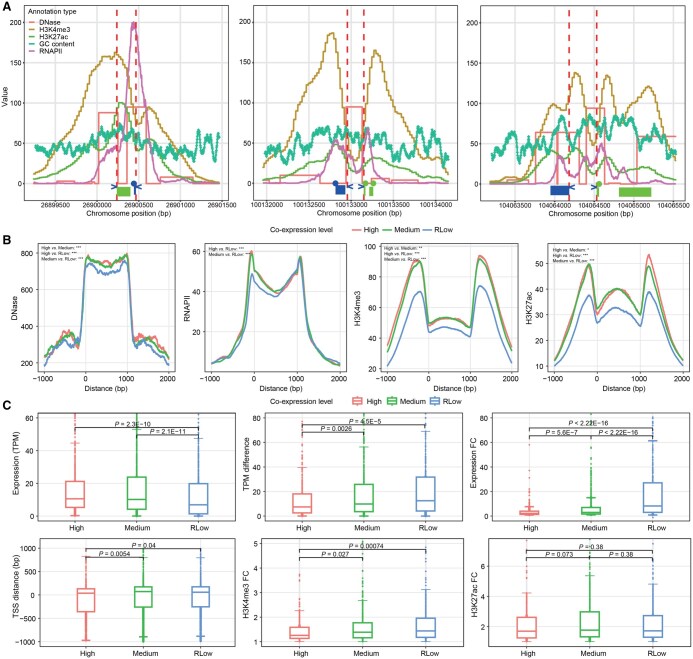
Epigenetic regulatory features of co-expressed DPGs **A**. An illustration of diverse regulatory signals in DPGs. Regulatory signals are color-coded. The two vertical red dashed lines depict TSSs of the two genes; the blue arrows indicate transcription directions. The green rectangle represents the 5′-UTR of genes on the positive strand, and the blue rectangle represents the 5′-UTR of genes on the negative strand. The colored dot signifies the start site of the first coding DNA. **B**. Average curves illustrating the DNase, RNAPII, H3K4me3, and H3K27ac signals of DPGs at different co-expression levels. The curve distributions, predominantly corresponding to the groups ranging from high to RLow, mostly display positive correlations. **C**. Box plots illustrating characteristics of DPGs at different co-expression levels, including expression level (TPM), expression difference, FC in expression, distance between TSSs, FC in H3K4me3 signal intensities, and FC in H3K27ac signal intensities. The first five box plots only show *P* values that indicate significant inter-group differences, while the final box plot of H3K27ac shows no inter-group differences. Statistically significant differences are denoted as follows: *, *P* < 0.05; **, *P* < 0.01; ***, *P* < 0.001. Mann–Whitney U test was used to compute the difference in (B and C). RNAPII, RNA polymerase II; TSS, transcription start site; RLow, relatively low; TPM, transcripts per million; FC, fold change.

To provide high-resolution views on regulatory mechanisms for DPGs, we incorporated four distinct epigenomic signal types, DNase, RNAPII, H3K4me3, and H3K27ac, along with sequence features such as GC content, using a sliding window approach (**Figure 4**A). High levels of RNAPII and DNase signals between the divergently transcribed genes of a DPG indicate increased chromatin accessibility, whereas H3K4me3 and H3K27ac signals highlight actively transcribed regions. These epigenetic marks reveal a regulatory landscape within the shared intergenic sequence of DPGs.

To find unique regularity characteristics of highly co-expressed DPGs, we grouped all DPGs into three levels based on co-expression correlation coefficients ranging from high to relatively low (RLow) and obtained mean curves for each epigenetic signal within each group. We observed positive correlations, in most cases, between co-expression levels and values of the four epigenetic signals (*P* < 0.05, Mann–Whitney U test; Figure 4B). As these signals signify active transcription, we further analyzed the expression disparity between the groups. Our results demonstrate that highly co-expressed DPGs exhibit a distinct set of characteristics. First, when compared to other DPGs, they show higher enrichment of DNase and RNAPII signals, as well as histone modifications, such as H3K4me3 and H3K27ac. Second, the genes within these DPGs not only exhibit higher expression levels but also show more comparable expression levels among themselves. Last, they have shorter or more overlapping inter-TSS distances compared to those of the other two groups (*P* < 0.05, Mann–Whitney U test; Figure 4C).

### Sequence spacing influences DPG co-expression modes

**Figure 5:**
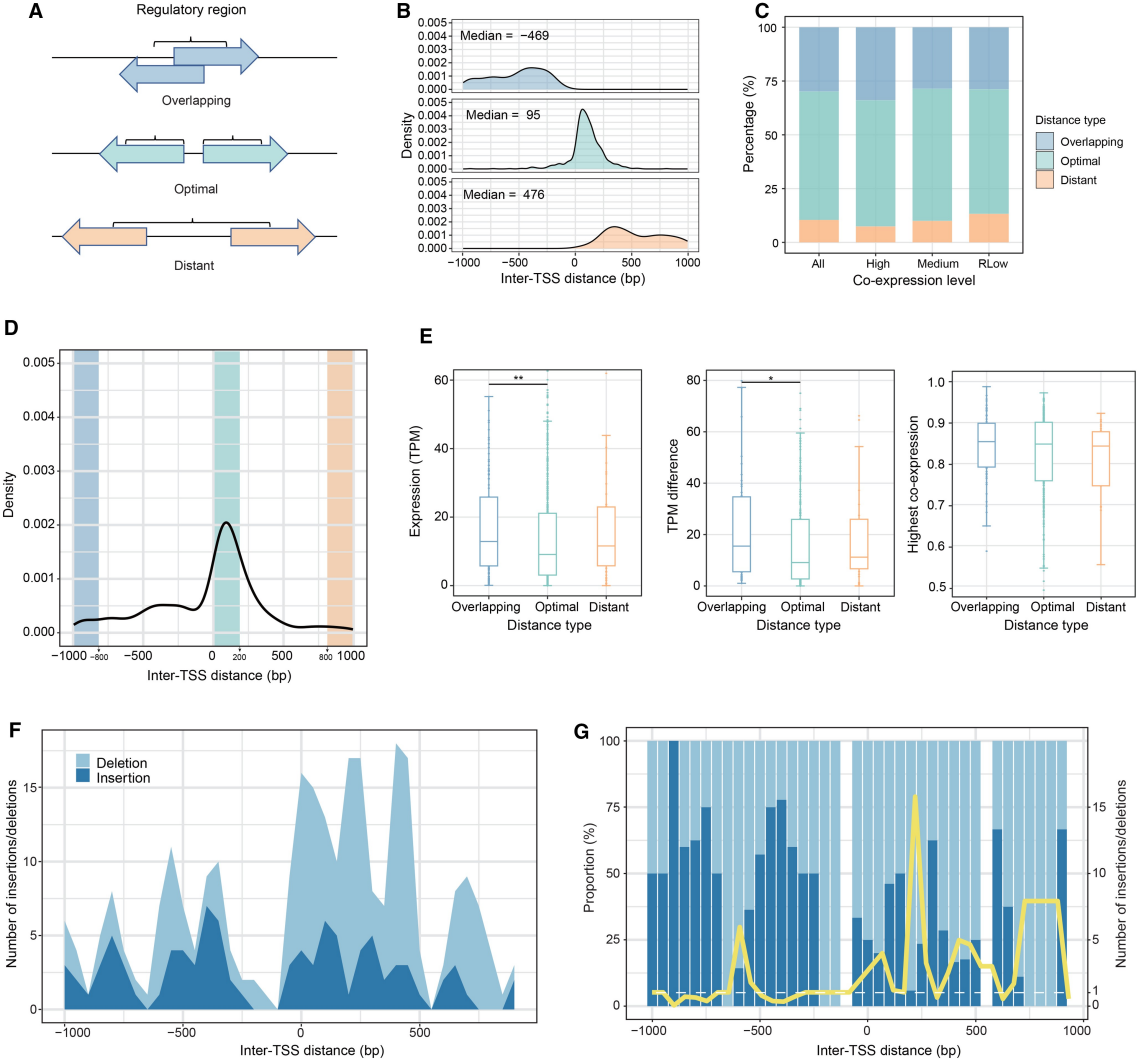
Sequence spacing within the regulatory regions of DPGs affects sequence stability and co-expression **A**. An illustration for inter-TSS distance or regulatory sequence spacing of DPGs. The spacing features are qualitatively classified into three basic types: overlapping, optimal, and distant. **B**. A diagram illustrating inter-TSS distances varying among DPGs in the three groups**. C**. Proportion of three types of DPGs in different co-expression level groups. **D**. Relative abundance of DPGs in the three types. **E**. Box plots illustrating expression level (TPM), expression difference, and co-expression of the three DPG types. The groups for comparisons are highlighted in (D). Mann–Whitney U test was used to compute the difference (*, *P* < 0.05; **, *P* < 0.01). **F**. Number of filtered indels plotted against the inter-TSS spacing. DPGs are partitioned based on inter-TSS distance with each data bin composed of 50-bp sequences. **G**. Proportion of insertions and deletions in each bin. The yellow curve shows the deletion/insertion ratio, and the number of insertions is set to greater than 1 for calculation. Inter-TSS, inter-transcription start site; indel, insertion and deletion.

H3K4me3 modification signals are markers for active promoters [35]. We further used peak positions to explore their regional distributions in DPGs. Overall, three distinct patterns were identified; each corresponds to a unique inter-TSS spacing distribution: overlapping, optimal, and distant (**Figure 5**A and B). The numbers of DPGs in the three groups were 390, 817, and 149, respectively. The distant group constitutes the smallest proportion among all DPGs, particularly diminished in highly co-expressed DPGs (Figure 5C), indicating a disadvantage in co-expression when the paired genes are too far apart from each other. In contrast, the overlapping and optimal groups of highly co-expressed DPGs are higher in proportion. The overlapping DPGs appear to be highly expressed individually and strongly co-expressed, albeit with greater differences in expression levels among the pairs (Figure 5D and E). This suggests that promoter space competition may occur when the two genes are highly overlapped or crowded spatially.

Therefore, together with results from epigenetic signals and expression variables, our analyses reveal that the regulatory pattern of the co-expressed DPGs follows three distance-sensitive modes. First, when inter-TSS distances are appropriately situated within a 95-bp window in median, the majority of these DPGs, *i.e.*, the optimal group, exhibit less biased co-expression and lower expression levels, and both are attributable to the shared open chromatin status and enriched *cis*-regulatory elements. Second, for DPGs with highly overlapping inter-TSS distances (with a median of −469 bp and extending to −1000 bp), the overlapping group appears to be highly expressed individually and more strongly co-expressed, albeit with greater differences in expression levels among the pairs. As inter-TSS distances increase (a median of 976 bp and extending to 1000 bp), the distant DPGs become separated, and their expression becomes less synchronized (*P* < 0.05, Mann–Whitney U test; Figure 5E).

To confirm genetic stability of the three DPG grouping schemes, we analyzed insertions and deletions (indels) in the inter-TSS sequence spacing across different DPG groups, using data from the expanded 1000 Genomes Project [36]. When the indels are plotted in a 50-bp sliding window, the selective trends are clearly demonstrated (Figure 5F and G). There are relatively more deletions in the optimal and distant DPGs, indicating that deletions contribute more substantially to the overall indels in these two types. The opposite trend is observed for the overlapping group, where higher proportions of insertions are observed. Additionally, the distribution of insertion events appears relatively uniform other than the obvious peak-and-valley patterns that are attributable to nucleosome positioning. Taken together, these results suggest that inter-TSS spacing between DPGs influences sequence stability and gene expression variability. Deviations in TSS spacing within DPGs lead to more sequence-space-altering or sequence-length-sensitive mutations.

With no major differences in the numbers or types of TF-binding sites among the three groups, we identified two clusters of TFs that are commonly shared among DPGs (Figure S2A). One functions in cell cycle monitoring, including G1/S phase transition and transcription activity, and the other involves epigenetic regulation of gene expression (Figure S2B and C). Taking vcDPGs as examples, by selecting the three most co-expressed TFs for each DPG as the core TFs, we showed a regulatory network composed of *ZNF263*, *ZNF134*, *SP2*, *SP3*, and *VEZF1*, as the most frequently shared TFs (Figure S2D). These results suggest a coordinated regulatory mechanism for DPGs in critical biological processes, such as cell cycle progression and chromatin remodeling. Notably, a higher number of co-expressed TFs are observed in TS co-expression of DPGs, whereas generally co-expressed DPGs do not show this pattern (Figure S2E). This result suggests that a greater number of TFs are required as cofactors for the complex, TS co-expression of DPGs.

### Conservation of primate-associated DPGs suggests strong function-driven convergent evolution

**Figure 6:**
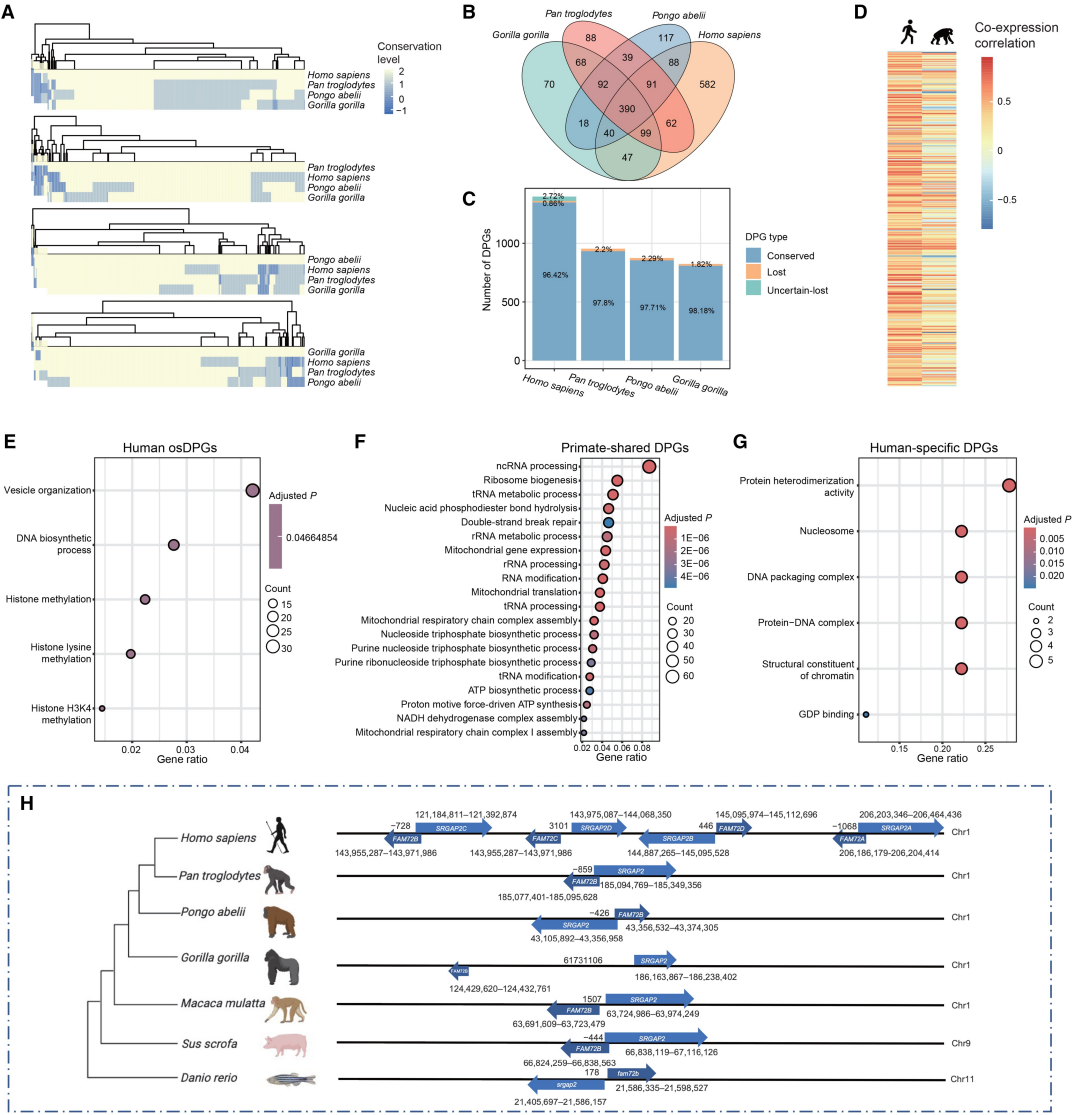
Conservation and specificity of human and primate-associated DPGs **A**. Heatmaps showing conservation of DPGs across the four primate species. Each for a species is shown in the first row. The darker color indicates lower conservation. The four values, ranging from 2 to −1, represent the four conservation forms as outlined in Figure 1, representing a conservation scale from high to low. The light blue block denotes the osDPGs in each species. **B**. Venn diagram depicting shared and unique DPGs among the four species. **C**. Bar plot illustrating the numbers of conserved and lost DPGs for each species. Conserved and lost groups denote DPGs that are either shared or have lost the orientations in at least one of the two counterparts of a DPG among the four primates, respectively. The human DPGs that are novel genes lacking annotation or repeatedly annotated at close positions are classified as “uncertain-lost” DPGs. The proportion of conserved DPGs in other three primates are similar (∼ 98%). **D**. Co-expression correlation coefficients of human osDPGs compared with their ortholog pairs in chimpanzee. **E**.–**G**. Functional enrichment of human osDPGs (E), primate-shared DPGs (F), and human-specific DPGs (G). **H**. An illustration of the human-specific DPGs, the *SRGAP2B–FAM72D* DPG family, and arrangements of their orthologous counterparts among other species. osDPG, orientation-specific DPG.

Since DPGs show diverse lineage-associated patterns during evolutionary processes among vertebrates, we focused on four representative species within the Hominidae family, *Homo sapiens*,* Gorilla gorilla*,* Pan troglodytes*, and *Pongo abelii*. Using a straightforward sequence alignment to map gain-and-loss events of lineage-associated and species-specific DPGs, we classified them into the four diversified types based on orthology and orientation. Despite the fact that DPGs of the four species are mostly shared at different degrees, there is still a set of orientation-specific DPGs (osDPGs), *i.e.*, those that have lost their original orientations or exhibit altered spacing. From a gene-to-function point of view, lineage-associated DPGs are undoubtedly diverse in an absolute sense, especially between the pairs, but rather narrow in principle toward synchronization of core molecular mechanisms and cellular processes (**Figure 6**A–D; Table S7). This point is further highlighted by a genome sequence alignment of human and chimpanzee in multiple regions where functional enrichments are largely associated with replication (such as DNA synthesis) and transcriptional regulation (such as histone methylation) (Figure 6E). In addition, the 390 highly-conserved Hominidae-specific DPGs, at both gene and orientation levels, are enriched in functions of post-transcriptional processes (such as ncRNA processing) and translation processes (such as ribosome biogenesis) (Figure 6F). Furthermore, 12 human-specific DPGs appear to be best enriched in post-translational processes, such as protein heterodimerization and chromatin construction (Figure 6G).

Employing the macaque as a common ancestor, we further investigated the origin of interspecific sequence variations of the human osDPGs. The difference in osDPGs between humans and chimpanzees originates from both deletions in humans and insertions in chimpanzees within the inter-TSS regions of DPGs (Figure S3A). The insertions in chimpanzees display an overrepresentation of repeat sequences of long interspersed nuclear elements (LINEs) and short interspersed nuclear elements (SINEs) (Figure S3B), with L1 and Alu being the most predominant types within each category (Figure S3C and D).

Several human-specific DPGs have been identified as playing pivotal roles in human development. Among these, the *SRGAP2–FAM72* gene pair was uniquely duplicated in human, leading to a total of four copies due to two gene duplication events, whereas this DPG remains single-copy in other vertebrate lineages (Figure 6H). Studies have shown that these DPGs are closely related to the development of nervous systems, such as the central cortex [37,38]. Also, human-associated DPGs play fundamental regulatory roles, such as *TUG1* as a Notch-regulated gene [39].

### Human-associated DPGs show diverse distribution within and among human populations

The T2T-CHM13 genome of European origin and the T2T-YAO genome of the Chinese population have provided precise and comprehensive gene annotations for different populations. By comparing the genomes from different populations, we identified a group of distinct population-specific DPGs (psDPGs). The results show that 55 and 357 DPGs are unique to YAO and CHM13, respectively (Figure S4A; Table S8). This finding indicates that DPGs not only exhibit unique patterns across different species but also display variations among populations. To assess the co-expression of psDPGs, we analyzed the RNA-seq data of healthy Chinese individuals obtained from the Genome Sequence Archive (GSA: HRA004352) [40]. By mapping the RNA-seq data to each reference genome and calculating the Pearson correlation coefficients, we constructed a co-expression heatmap of these DPGs. The results reveal differences in co-expression among psDPGs (Figure S4B). These variations may represent potential mechanisms contributing to the distinctive traits observed in each population.

## Discussion

Most, if not all, genes are non-randomly partitioned over chromosomes and chromatin territories. In general, genes tiling along chromosomes are selected for replication and subsequent cell differentiation, whereas those organized within chromatin territories are selected for transcription and cellular functions. In these regards, DPGs are an organizationally-diverse and functionally-diverse class of co-expressed genes, and studying their evolution is essential for understanding their mechanistic details and for classifying them in lineage-associated and functionally-correlated ways [3,41–49]. In this study, we demonstrate these points of view by focusing our analyses on coarsely selected vertebrate lineages with a human-centric framework, as our previous investigations have suggested that evolutionary selectivity is biased between invertebrates (*e.g.*, *Drosophila*) and vertebrates [3]. We further suggest that future DPG studies should pay special attention to systematic annotations of vertebrate DPGs, as others have proposed for vertebrate biology and human disease studies [6,13]. In addition, the evolution of DPGs across eukaryotic lineages is both divergent and convergent in nature. On larger evolutionary scales, highly conserved DPGs are mostly involved in highly conserved cell cycle or spatiotemporal-parameter-sensitive processes, through inventions of, by and large, direct binding or interaction for both relevant and irrelevant pathways and networks [4,10,50], whereas for closely related species or genera, many novel species-associated or lineage-associated DPGs appear to be generated for tissue-specific or organ-specific functions.

It is rather surprising that our comparative studies among closely related primates and between human populations reveal striking differences in DPGs, and such a result suggests that the in-and-out relationship created by forming and destroying DPGs is frequent in species evolution. Comparison between human and chimpanzee reveals that human psDPGs arise mostly from biased sequence variations both in type (such as deletions *vs*. insertions) and in number (such as more deletions in the human genome and more insertions in the chimpanzee genome during such a rather short evolutionary time scale). Moreover, the insertion of Alu and L1 transposable elements in chimpanzees contributed predominantly to the divergence between orthologs. Koyanagi et al. have demonstrated that the emergence of DPGs involves exploiting pre-existing genes, showing consistency with our results [6]. Furthermore, we also identified that a portion of DPGs emerges specifically in certain species due to segmental duplications or gene duplications (Figure 6H). Furthermore, DPGs exhibit differences between human populations. Our comparison of the Chinese (T2T-YAO) and European (T2T-CHM13) reference genomes highlights the existence of variability and specificity among DPGs. These distinctive population-associated characteristics of DPGs may provide novel insights into how genomes should be analyzed and what targeted therapeutics should be developed for future medicine.

Evolution with a limited repertoire of genes has resulted in complex life forms and multi-tracked holovivological systems, whose molecular details are believed to be involved in five distinct tracks: information, operation, homeostasis, compartmentalization, and plasticity [42,51]. On the informational track, DPGs can be mapped out systematically based on high-quality genome assemblies, and such a mapping process can be extended in one dimension, *e.g.*, to construct databases for DPGs of all species, and in the other dimension, *e.g.*, to map DPGs between protein-coding and non-protein-coding gene pairs. Although the ultimate goal of such studies is to divide all genes either into clusters or as individually regulated units, efforts toward the final goal have to be stepwise. On the operational track, the regulatory mechanisms governing DPGs in different cellular processes are to be compared to those of non-clustered genes. Based on our current study, we are able to draw four basic conclusions. First, there have not been novel mechanisms in regulating DPG expression other than better coordinated expression at either the same or uneven levels. Second, there have not been many unique or novel regulatory elements and mechanisms within the inter-TSS sequence spaces other than the enrichment of certain *trans*-regulatory TF classes and *cis*-regulatory sequence variations that are sensitive to sequence lengths, which are measurable based on indel events according to population genetic conception. Third, the frequency distribution of indels within the inter-TSS sequences also shows a strong association with nucleosome phasing or space occupation, as shown in our previous work [52], and the phenomenon supports the two previous notions. Fourth, the length variability of the inter-TSS distances among the three types of DPGs confirms that it is not only selected but also has a tendency to maintain an optimal length for the best fit of their *trans*-regulatory elements. Interestingly, a similar trend was observed in small introns with optimal lengths, as highlighted in our previous work [53–57]. Our final notion is to emphasize that our study indicates the necessity for further investigation of DPGs on the other three holovivological tracks where their functional relevance to cell cycle timing and brain association is to be further inspected.

In conclusion, our discoveries underscore the essential role of DPGs in the human genome, presenting new avenues for understanding their contribution to genetic regulation and evolutionary biology. The observed patterns of conservation and selection suggest that DPGs are not only structural elements but also pivotal in maintaining genomic integrity and functionality. Future research should continue to explore the regulatory mechanisms and evolutionary trajectories of DPGs, which will provide deeper and more detailed insights into their biological significance and potential applications in genomics and medicine.

## Materials and methods

### Species selection and data collection

In order to conduct a conservation analysis of human DPGs in vertebrates, an additional 45 species were selected across a range of evolutionary scales, including fish, amphibians, reptiles, mammals, primates, and birds. This was done following a quality screening with contig N50 values greater than 15 kb (Table S2). All species were from the Ensembl database (v108) [58], including DNA sequences, protein sequences, and general feature format version 3 (gff3) files for further analysis.

### Epigenetic data collection

The epigenetic data used in this study were sourced from the Encyclopedia of DNA Elements (ENCODE) project [59], accessed via the University of California Santa Cruz (UCSC) Table Browser [60] and the file transfer protocol (FTP) site. The datasets for H3K4me3 (wgEncodeRegMarkH3k4me3) and H3K27ac (wgEncodeRegMarkH3k27ac) were obtained from UCSC. Six non-disease cell lines were retained from these two datasets, including the lymphoblastoid cell line (GM12878), H1 human embryonic stem cell (H1-hESC), human skeletal muscle myoblast (HSMM), human umbilical vein endothelial cell (HUVEC), normal human epidermal keratinocyte (NHEK), and normal human lung fibroblast (NHLF). The signal values were calculated and averaged across the six cell types. The DNase dataset (wgEncodeRegDnaseClustered) was also downloaded from UCSC. RNAPII data were obtained from the ENCODE portal [61], with three cell types selected for analysis: GM12878, H1-hESC, and HUVEC. The corresponding accession numbers of these three datasets are ENCFF322DRU, ENCFF942TZX, and ENCFF696JRV.

### Orthologous gene identification

The orthologous gene relationships between species were identified by OrthoFinder (v2.5.4) [62] with default parameters. Protein sequences from all species were used as input, and for genes with multiple transcripts, the longest transcript was retained. Orthologous genes provided by the Ensembl database were included as a complement in the comparative analysis of invertebrates and Hominidae species, and the data were retrieved via Ensembl BioMart [63]. With regard to the discrepancy between the two orthologous datasets, both were preserved. The combination method and data format are in reference to the homologous gene database (HGD) [64].

### Identification and comparison of DPGs

To identify DPGs, we screened protein-coding genes in the gene annotation files of each species. We found gene pairs that met the requirements of opposite transcription directions on the same chromosome and a distance between the TSSs of less than 1 kb. To construct a cross-species conservation map of DPGs, we searched for the corresponding orthologous genes in other species. We classified all DPGs into four types of conservation patterns: (1) highly conserved, referring to DPGs that are conserved across the species being compared and have orthologous genes maintaining the same orientation; (2) conserved, referring to DPGs that are conserved across the species but have one of the paired genes losing its original orientation; (3) lost single, referring to DPGs that lose one of the paired genes in an orthologous counterpart; and (4) lost both, referring to DPGs where both genes are missing at the original location in the orthologous counterpart. The R package pheatmap was used to plot the conservation values of all DPGs across species. Hierarchical clustering was performed based on a complete linkage method to cluster rows of species. The comparison between different human reference genomes followed a similar approach to cross-species mapping: all DPGs in different reference genomes were identified and mapped to each other based on gene symbols. Unmapped DPGs were referred to as psDPGs for further co-expression comparisons.

### HK genes in DPGs

The list of HK genes was downloaded from a previous study [28]. The HK genes within DPGs were determined by matching the HK gene list with the DPGs. The bitr function of the R package clusterProfiler [65] was used for gene ID conversion. DPGs containing at least one HK gene were defined as HK-DPGs.

### Functional enrichment analysis of DPGs

Gene functional enrichment analysis was conducted using the R package clusterProfiler [65]. The BH method was employed to adjust the *P* values, with a significance threshold of adjusted *P* < 0.05. The Database for Annotation, Visualization and Integrated Discovery (DAVID; v6.8) [66] was employed to perform functional enrichment analyses across multiple categories, including GO terms (*i.e.*, BP, CC, and MF) and pathways (*i.e.*, Kyoto Encyclopedia of Genes and Genomes), for candidate gene lists using *H. sapiens* as the background, with significance determined by a two-tailed Fisher’s exact test followed by multiple testing correction (*P* < 0.05).

### PPI and RNA–protein interactions

The PPI data were retrieved from the Search Tool for the Retrieval of Interacting Genes/Proteins (STRING) [67] database using the medium-confidence filtering threshold. In Figure 2D, only the two main clusters were retained, and the dispersed nodes were reduced. The combined interaction score between the genes was used to calculate the edge thickness. The data on RNA*–*protein interactions were obtained from the RNA interactome (RNAInter) database (v4.0) [68], which were filtered with a high-confidence threshold of score > 0.2. The networks were constructed using the Cytoscape software (v3.10.0) [69].

### Co-expression analysis of DPGs

Gene expression data were obtained from the Genotype-Tissue Expression (GTEx) Portal [70]. Samples were quality controlled using RNA integrity number (RIN) > 6. After filtering, only tissues with more than 20 samples were retained (Figure S1D). Transcripts per million (TPM) values of the samples in all tissues were obtained and then natural log-transformed [ln (TPM + 1)]. The TPM value of each gene in a specific tissue was represented by the median TPM value across all samples. For each DPG, the Spearman correlation coefficient between the expression levels of the pairs was computed across all samples in each tissue using the spearmanr function of the SciPy package (v1.11.2) [71]. The resulting correlation coefficients were organized into a matrix and plotted as a heatmap using the pheatmap package in R.

A filtering process was applied to identify co-expressed DPGs in each tissue. DPGs with a co-expression coefficient greater than 0.8 and a *P* value less than 0.05 were retained. The fold change (FC) in expression for each DPG was calculated by dividing the expression of the highly expressed gene by the expression of the lower expressed gene in the tissue with the highest co-expression. The criterion for identifying the generally co-expressed DPGs was a co-expression coefficient greater than 0.7 in more than 70% of the analyzed tissues.

The RNA-seq dataset GSE127898 [72] was used to examine co-expression variation of psDPGs between humans and chimpanzees in Figure 6D, following a similar process.

### Epigenetic characteristic analysis

Regulatory profiles for each pair of DPGs were visualized by the R package ggplot2. For each DPG, data were processed based on its specific genomic positions. The DNA methylation signals were amplified by a factor of 20, while the DNase values were divided by 10 for better display. GC content was calculated by sliding windows, with a window size of 50 bp and a step of 3 bp. Regulatory regions were identified by analyzing the relative positions of the TSSs and the peaks of H3K4me3. The peak positions were identified using the find_peaks function from the scipy.signal module.

The DPGs were classified into three groups based on the highest co-expression correlation coefficient observed among all tissues: high (> 0.9), medium (0.8–0.9), and RLow (< 0.8). For each group, the average signal profiles were generated for four signal types: DNase, RNAPII, H3K4me3, and H3K27ac. For each category, the distance between the TSSs of two genes in DPGs was normalized and uniformly scaled to 1000 bp. The value at each position was averaged for all DPGs, resulting in a single value for each position.

### Identification of shared TFs among DPGs

The TF motif data were obtained from the HOmo sapiens COmprehensive MOdel COllection (HOCOMOCO) database (v11) [73], which contain 769 full human TF motifs. The data were downloaded in MEME format from the MEME Suite web server (v5.5.4) [74]. The binding site analysis was performed by using the find individual motif occurences (FIMO) [75] command-line version from the MEME Suite. The analysis was based on motif file inputs along with sequence files of regulatory regions, with the selected parameter “--norc” to analyze the transcription factor binding sites (TFBSs) on the strand where the gene resides. The co-expression of shared TFs was analyzed by the co-expression analysis method employed for DPGs. A filtering criterion with a correlation coefficient greater than 0.8 was used to identify co-expressed TFs as shown in Figure S2A.

### Identification of sequence variations in DPGs

Multiple sequence alignment was performed on the intergenic regions of human osDPGs and the corresponding orthologous regions in chimpanzee and macaque. The genomic sequences of the macaque were downloaded from the Ensembl database (genome assembly GCA_003339765.3). For the genomic sequences of the chimpanzee that do not align with the human genome, they were compared with the macaque genome. Regions shared by chimpanzee and macaque but absent in human were defined as human-lost regions, while regions absent in macaque were defined as chimpanzee insertions. RepeatMasker [76] was used to analyze the repeat sequences in these specific regions of the chimpanzee genome.

The indels within the inter-TSS sequences were derived from the 1000 Genome Project [36]. The indels were filtered based on a frequency threshold of greater than or equal to 0.005 and a length change of greater than or equal to 2 bp. All DPGs were grouped based on the inter-TSS distance of DPGs, ranging from −1 kb to 1 kb in 50-bp bins.

### Statistical analysis

All data were analyzed using Python (v3.11.4), with the Pandas and NumPy packages utilized for data analysis, computation, and preprocessing. The SciPy package (v1.11.2) [71] was used to calculate differences between groups using the Fisher’s exact test for small groups and the Chi-squared test for large groups (*n* > 40), both with *P* < 0.05 as the criterion. Differences in distributions were tested using the Mann–Whitney U test in R (v4.3.1).

## Code availability

The code for DPG identification has been submitted to BioCode and compiled into a lightweight toolkit, at the National Genomics Data Center (NGDC), China National Center for Bioinformation (CNCB) (BioCode: BT007623), which is publicly accessible at https://ngdc.cncb.ac.cn/biocode/tool/BT7623.

## CRediT author statement

**Guangya Duan:** Data curation, Methodology, Formal analysis, Visualization, Writing – original draft, Writing – review & editing. **Sisi Zhang:** Methodology, Resources. **Bixia Tang:** Methodology. **Jingfa Xiao:** Methodology, Resources, Writing – review & editing. **Zhang Zhang:** Methodology, Resources, Writing – review & editing. **Peng Cui:** Conceptualization, Methodology, Resources, Writing – original draft, Writing – review & editing, Supervision. **Jun Yu:** Conceptualization, Methodology, Resources, Writing – original draft, Writing – review & editing, Supervision. **Wenming Zhao:** Conceptualization, Methodology, Resources, Writing – original draft, Writing – review & editing, Supervision. All authors have read and approved the final manuscript.

## Competing interests

The authors have declared no competing interests.

## Supplementary Material

qzaf058_Supplementary_Data
