# Supplementary material for: Lineage-associated Human Divergently-paired Genes Exhibit Structural and Regulatory Characteristics
Source: Genomics Proteomics Bioinformatics. 2025 Jun 26;23(4):qzaf058. doi: 10.1093/gpbjnl/qzaf058 (PMC12672016; doi:10.1093/gpbjnl/qzaf058)
Supplement: qzaf058_Supplementary_Data [file qzaf058_supplementary_data.zip › Figure S3.pdf]

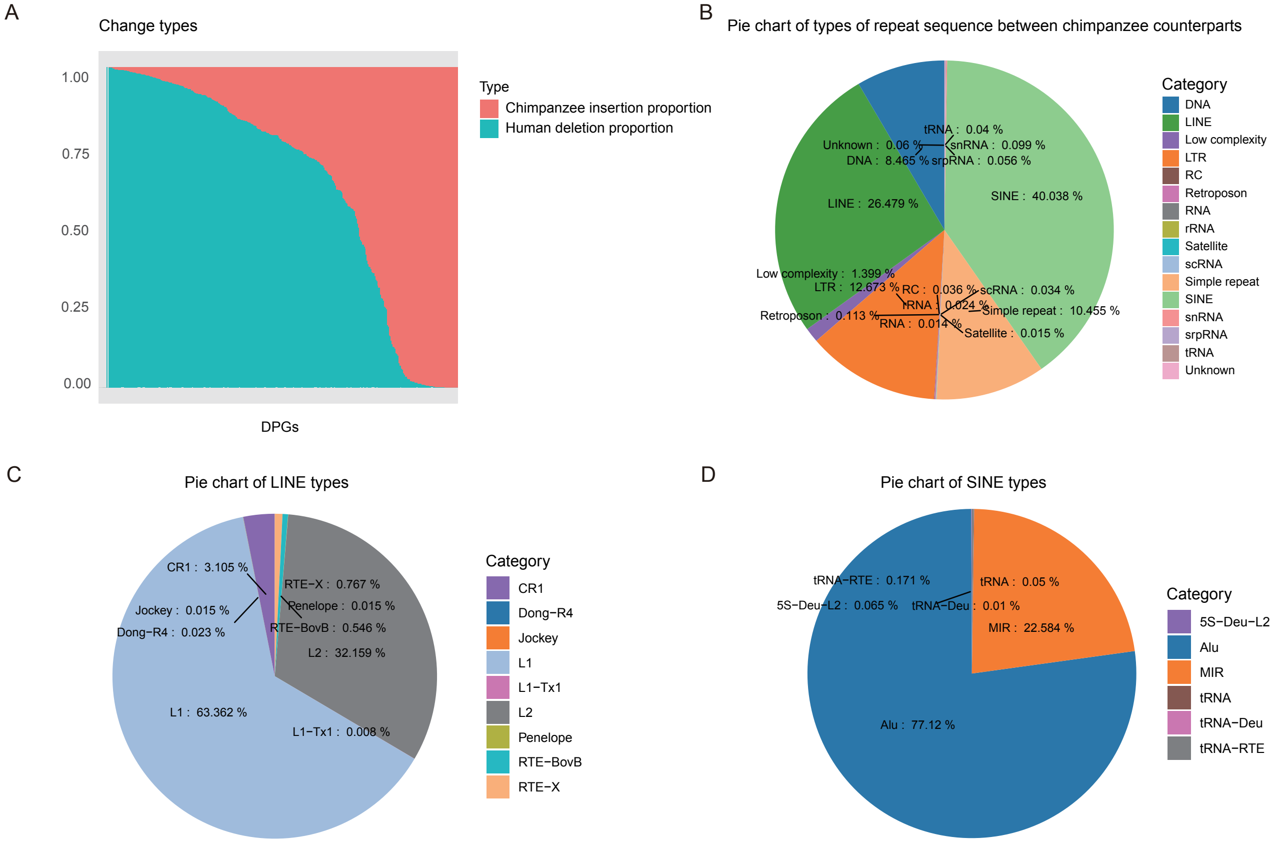

A
Change types
1.00
0.75
0.50
0.25
0.00
DPGs
Type
Chimpanzee insertion proportion
Human deletion proportion
B
Pie chart of types of repeat sequence between chimpanzee counterparts
tRNA : 0.04 %
Unknown : 0.06 %
snRNA : 0.099 %
DNA : 8.465 %
srpRNA : 0.056 %
SINE : 40.038 %
LINE : 26.479 %
Low complexity : 1.399 %
LTR : 12.673 %
RC : 0.036 %
scRNA : 0.034 %
rRNA : 0.024 %
Simple repeat : 10.455 %
Retroposon : 0.113 %
RNA : 0.014 %
Satellite : 0.015 %
Category
DNA
LINE
Low complexity
LTR
RC
Retroposon
RNA
rRNA
Satellite
scRNA
Simple repeat
SINE
snRNA
srpRNA
tRNA
Unknown
C
Pie chart of LINE types
CR1 : 3.105 %
RTE−X : 0.767 %
Penelope : 0.015 %
Jockey : 0.015 %
RTE−BovB : 0.546 %
Dong−R4 : 0.023 %
L2 : 32.159 %
L1 : 63.362 %
L1−Tx1 : 0.008 %
Category
CR1
Dong−R4
Jockey
L1
L1−Tx1
L2
Penelope
RTE−BovB
RTE−X
D
Pie chart of SINE types
tRNA−RTE : 0.171 %
tRNA : 0.05 %
5S−Deu−L2 : 0.065 %
tRNA−Deu : 0.01 %
MIR : 22.584 %
Alu : 77.12 %
Category
5S−Deu−L2
Alu
MIR
tRNA
tRNA−Deu
tRNA−RTE
