# Supplementary material for: Lineage-associated Human Divergently-paired Genes Exhibit Structural and Regulatory Characteristics
Source: Genomics Proteomics Bioinformatics. 2025 Jun 26;23(4):qzaf058. doi: 10.1093/gpbjnl/qzaf058 (PMC12672016; doi:10.1093/gpbjnl/qzaf058)
Supplement: qzaf058_Supplementary_Data [file qzaf058_supplementary_data.zip › supplementary material captions.docx]

**Supplementary materials**

**Figure S1 Co-expression patterns of vcDPGs**

**A.** A heatmap of co-expressed vcDPGs. DPG and expressed tissue are shown in rows and columns, respectively. The *P* values of the Spearman correlation coefficient are denoted as follows: *, *P* < 0.05; **, *P* < 0.01; ***, *P* < 0.001. **B.** GO enrichment of generally co-expressed DPGs. **C.** PPI network of brain–testis co-expressed DPGs. **D.** Number of samples of each tissue after quality control.

**Figure S2 vcDPGs with shared co-expressed TFs among tissues**

**A.** A heatmap shows the shared TFs among DPGs. The blue color denotes shared transcription factor binding sites (TFBS) at regulatory regions of DPGs, whereas the yellow color does not. TFs (rows) and DPGs (columns) are grouped based on two-way hierarchical clustering. **B.** and **C.** Functional enrichment of the two groups of most shared TF clusters. **D.** A network depicts co-expressed DPGs and shared TFs. Green triangles and yellow circles represent DPGs and TFs, respectively. Only the top three co-expressed TFs are shown. **E.** A heatmap shows co-expressed TFs shared by vcDPGs across various tissues, with a filtering criterion of co-expression value of Spearman correlation coefficient > 0.8 and *P* value < 0.05. Stars and colors indicate co-expression of DPGs and the number of expression-shared TFs, respectively. TF, transcription factor.

**Figure S3 Comparison of inter-TSS sequence between human osDPGs and corresponding regions in chimpanzees**

**A.** The human osDPGs is compared to those of chimpanzee based on alignment to ancestor sequences referenced of the macaque genome. **B.** A pie chart illustrates the types of repetitive sequences in the region between orthologous counterparts of human osDPGs in chimpanzee. **C.** and **D.** Types of LINE and SINE insertions between orthologous genes of human osDPGs in chimpanzee. LINE, long interspersed nuclear element; LTR, long terminal repeat; RC, reverse complement; scRNA, single-cell RNA; SINE, short interspersed nuclear element; snRNA, small nuclear RNA; srpRNA, signal recognition particle RNA.

**Figure S4 A population-based analysis of different reference genomes**

**A.** A diagram shows DPGs shared or unique to each human reference genome. Both haplotypes of the YAO genome are included. **B.** A heatmap illustrates the co-expression of all psDPGs. Rows represent DPGs, and the annotation bar denote their population specificities. psDPGs, population-specific DPGs; YAO-mat, YAO maternal haplotype; YAO-pat, YAO paternal haplotype.

**Table S1 Lists of all protein-coding DPGs and vcDPGs involved in this study**

**Table S2 A list of vertebrate species involved in this study**

**Table S3 A list of human DPGs conserved in selected invertebrate species**

**Table S4 Lists of DPGs involved in protein****–protein, protein–DNA, and protein****–ion interactions**

**Table S5 All tissues and corresponding abbreviations involved in this study**

**Table S6 A list of DPGs that are commonly co-expressed among different tissues**

**Table S7 Lists of human osDPGs in types and unique human DPGs**

**Table S8 DPGs identified in the three reference genomes**
