# Supplementary figures and images for: Lineage-associated Human Divergently-paired Genes Exhibit Structural and Regulatory Characteristics
Source: Genomics Proteomics Bioinformatics. 2025 Jun 26;23(4):qzaf058. doi: 10.1093/gpbjnl/qzaf058 (PMC12672016; doi:10.1093/gpbjnl/qzaf058)

A

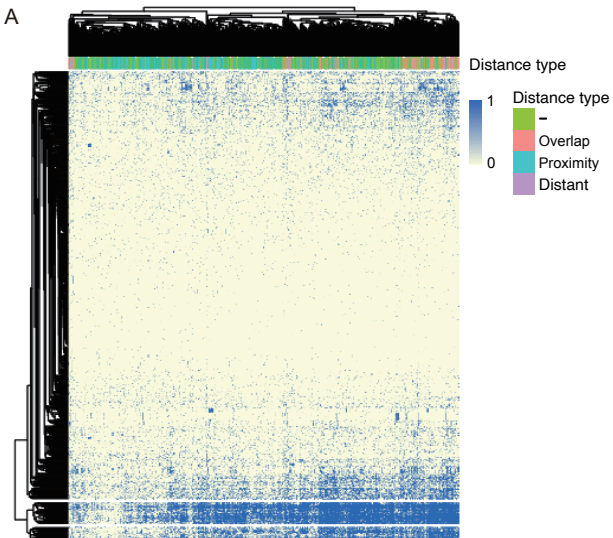

B

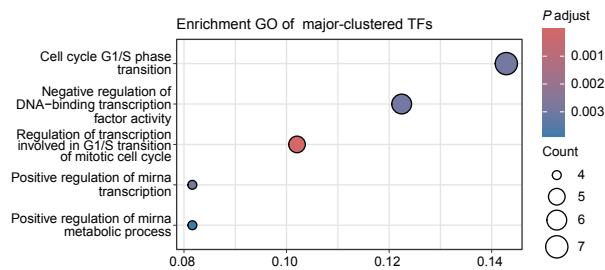

C

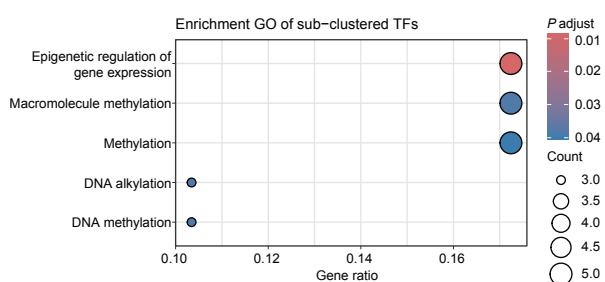

D

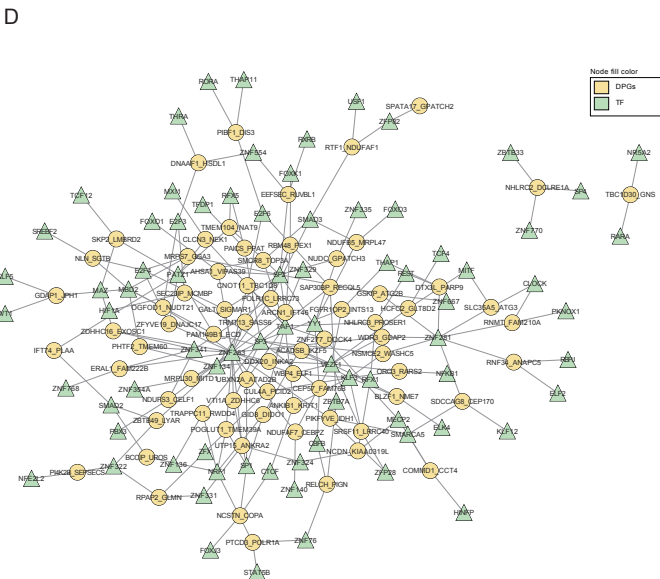

E

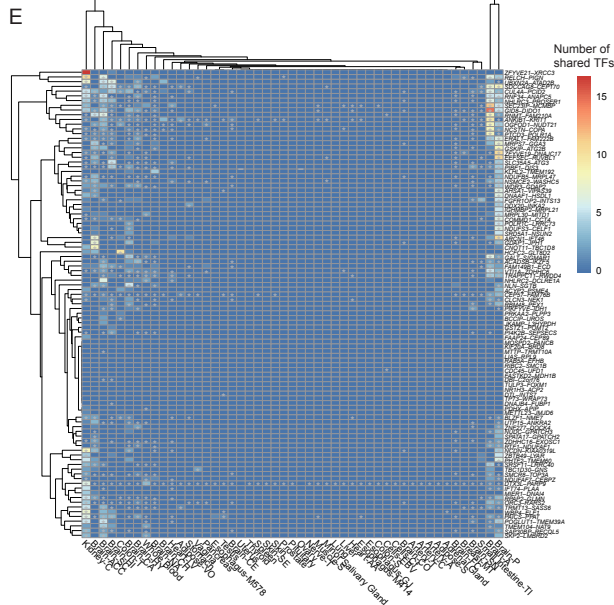

Supplement: qzaf058_Supplementary_Data [file qzaf058_supplementary_data.zip › Figure S2.pdf]

A

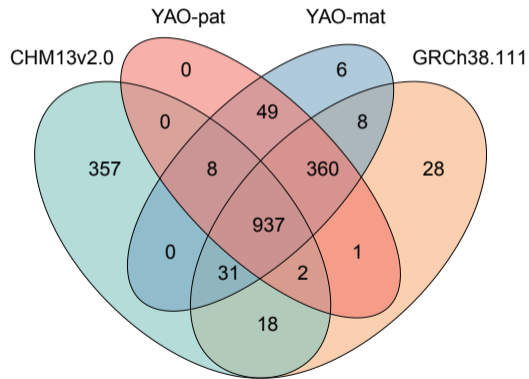

B

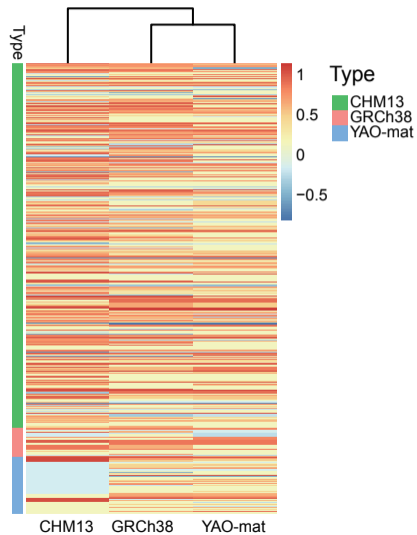

Supplement: qzaf058_Supplementary_Data [file qzaf058_supplementary_data.zip › Figure S4.pdf]
