# Supplementary material for: Lineage-associated Human Divergently-paired Genes Exhibit Structural and Regulatory Characteristics
Source: Genomics Proteomics Bioinformatics. 2025 Jun 26;23(4):qzaf058. doi: 10.1093/gpbjnl/qzaf058 (PMC12672016; doi:10.1093/gpbjnl/qzaf058)
Supplement: qzaf058_Supplementary_Data [file Figure S1.pdf › Figure S1.pdf]

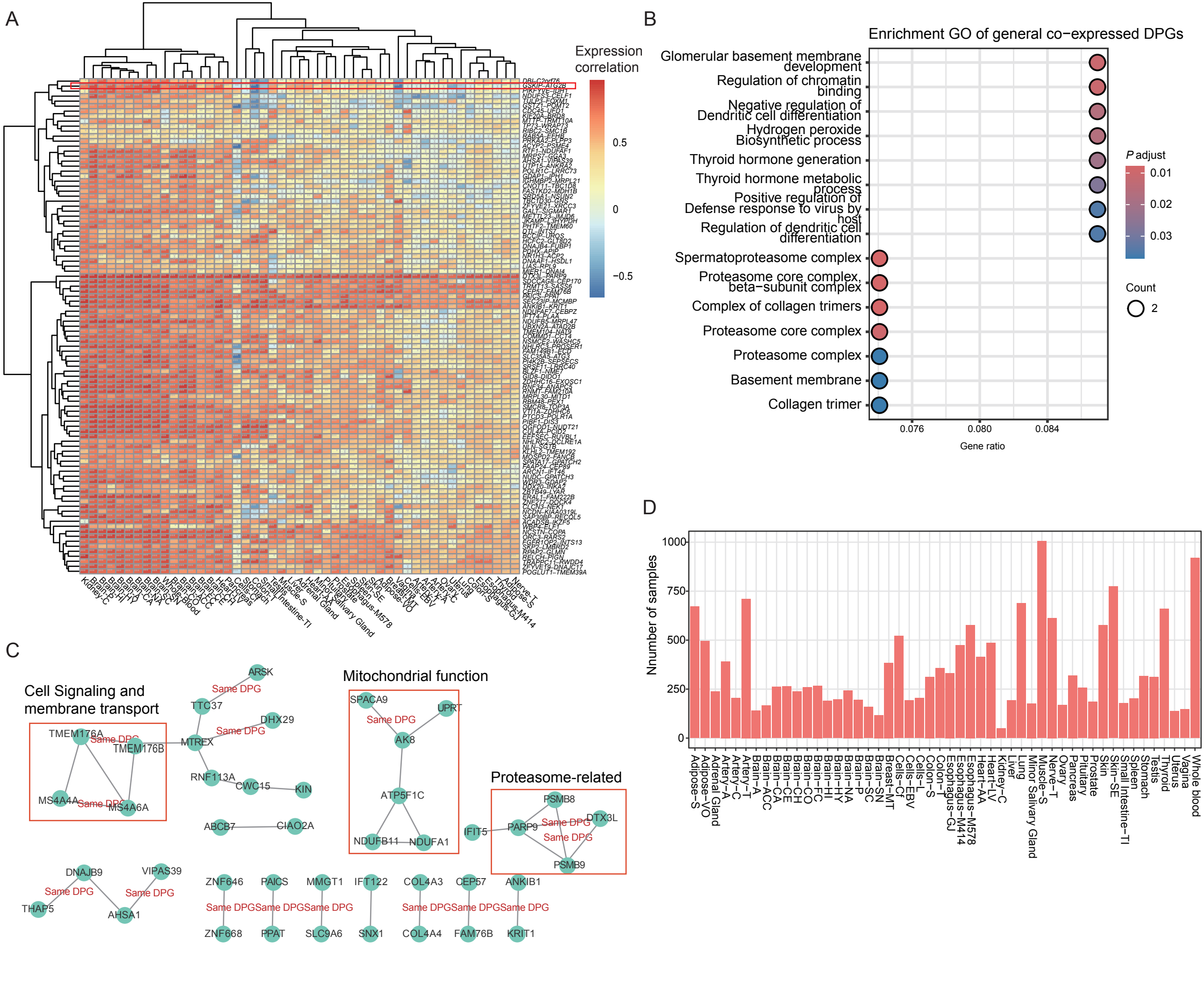
A
Expression correlation
0.5
0
−0.5
B
Enrichment GO of general co−expressed DPGs
Glomerular basement membrane development
Regulation of chromatin binding
Negative regulation of Dendritic cell differentiation
Hydrogen peroxide Biosynthetic process
Thyroid hormone generation
Thyroid hormone metabolic process
Positive regulation of Defense response to virus by host
Regulation of dendritic cell differentiation
Spermatoproteasome complex
Proteasome core complex, beta−subunit complex
Complex of collagen trimers
Proteasome core complex
Proteasome complex
Basement membrane
Collagen trimer
0.076
0.080
0.084
Gene ratio
P adjust
0.01
0.02
0.03
Count
2
C
Cell Signaling and membrane transport
Mitochondrial function
Proteasome-related
Same DPG
TMEM176A
TMEM176B
MS4A4A
MS4A6A
ARSK
TTC37
DHX29
MTREX
RNF113A
CWC15
KIN
ABCB7
CIAO2A
SPACA9
UPRT
AK8
ATP5F1C
NDUFB11
NDUFA1
IFIT5
PARP9
PSMB8
DTX3L
PSMB9
DNAJB9
VIPAS39
THAP5
AHSA1
ZNF646
ZNF668
PAICS
PPAT
MMGT1
SLC9A6
IFT122
SNX1
COL4A3
COL4A4
CEP57
FAM76B
ANKIB1
KRIT1
D
Nnumber of samples
0
250
500
750
1000
Adipose−S
Adipose−VO
Adrenal Gland
Artery−A
Artery−C
Artery−T
Brain−A
Brain−ACC
Brain−CA
Brain−CE
Brain−CH
Brain−CO
Brain−FC
Brain−HI
Brain−HY
Brain−NA
Brain−P
Brain−SC
Brain−SN
Breast−MT
Cells−Cf
Cells−EBV
Cells−L
Colon−S
Colon−T
Esophagus−GJ
Esophagus−M414
Esophagus−M578
Heart−AA
Heart−LV
Kidney−C
Liver
Lung
Minor Salivary Gland
Muscle−S
Nerve−T
Ovary
Pancreas
Pituitary
Prostate
Skin
Skin−SE
Small Intestine−TI
Spleen
Stomach
Testis
Thyroid
Uterus
Vagina
Whole blood
